# Association between Neighborhood Food Environment and Body Mass Index among Older Adults in Beijing, China: A Cross-Sectional Study

**DOI:** 10.3390/ijerph17207658

**Published:** 2020-10-20

**Authors:** Man Zhang, Wen Guo, Na Zhang, Hairong He, Yu Zhang, Mingzhu Zhou, Jianfen Zhang, Muxia Li, Guansheng Ma

**Affiliations:** 1 Department of Nutrition and Food Hygiene, School of Public Health, Peking University, No. 38 Xueyuan Road, Haidian District, Beijing 100191, China; zhangman@bjmu.edu.cn (M.Z.); wguo14@pku.edu.cn (W.G.); ziqingxuanping@126.com (N.Z.); hehairong_16@bjmu.edu.cn (H.H.); zhangyu30171026@163.com (Y.Z.); zmz6290@163.com (M.Z.); 13161522166@163.com (J.Z.); lmuxia91@126.com (M.L.); 2 Laboratory of Toxicological Research and Risk Assessment for Food Safety, Peking University, No. 38 Xueyuan Road, Haidian District, Beijing 100191, China

**Keywords:** food environment, obesity, body mass index, older adults

## Abstract

Objective: To investigate the association between the neighborhood food environment and body mass index (BMI) among Chinese older adults. Methods: A multi-stage stratified random sampling method was used to recruit participants from 12 communities in Beijing, China, in 2019. Participants (*n* = 1764, 1034 women) in this study were older adults aged 65 to 80. We collected the participants’ basic information, measured their height and weight, and calculated their BMI. Neighborhood food environments were measured by the density of and proximity to different food outlets using the Baidu Map Application Programming Interface. Adjusted multiple linear regression was performed to estimate the association between the food environment and BMI. Results: Participants had a mean age of 69.7 ± 4.32 years old and an average BMI of 26.3 ± 3.50 kg/m^2^. Among the three types of stores, convenience stores had the easiest access, followed by greengrocers and supermarkets. Sit-down restaurants had the best access among different restaurants, followed by Chinese fast-food restaurants, and western fast-food restaurants had the worst access. Easier access to greengrocers (β = 0.281, *p* < 0.001) and sit-down restaurants (β = 0.304, *p* < 0.001) was associated with higher BMI in the 250 m buffer zone. More supermarkets were associated with higher BMI in the 500 m buffer zone (β = 0.593, *p* < 0.001). Access to convenience stores was positively associated with BMI in the 800 m buffer zone (β = 0.057, *p* < 0.001). Better access to Chinese fast-food restaurants was associated with higher BMI (β = 0.071, *p* = 0.001), and better access to western fast- food restaurants was associated with lower BMI (β = −0.400, *p* < 0.001) in the 1000 m buffer zone. There was a negative association between the nearest distance to greengrocers and BMI (β = −0.004, *p* < 0.001). Conclusion: Although we found some significant associations between the neighborhood food environment and obesity, the current results are not strong enough to draw specific conclusions. Policymakers will need to rely on more evidence to derive concrete policy recommendations.

## 1. Introduction

Along with socioeconomic development and lifestyle changes, overweight and obesity rates in developed and developing countries have increased. In recent years, the rate of overweight and obesity among the elderly in China has risen rapidly [1]. According to survey data of the China Health and Retirement Longitudinal Study (CHARLS), the overweight rate increased from 26.14% in 2011 to 30.14% in 2015, and the rate of obesity increased from 9.58% to 10.75% [1]. Three studies conducted between 2017 and 2018 in Beijing, the capital of China, found that the overweight and obesity rates of the elderly over 65 were 46.64%, 64.96%, and 69.34%, respectively [2,3,4]. Increased BMI is an established risk factor for diseases, such as type 2 diabetes, cardiovascular diseases, and many cancers [5]. For every 5-unit increase in body mass index (BMI) above 25 kg/m^2^, overall mortality increases by 29%, vascular mortality by 41%, and diabetes-related mortality by 210% [6]. Obesity is associated with reduced earning potential and higher healthcare costs that may result in an economic burden on society [7]. In 2010, overweight and obesity imposed a substantial economic burden on China, responsible for 42.9% of the medical and non-medical yearly costs of the major non-communicable diseases (NCDs) in China, with amounting to 90.768 billion renminbi (RMB) [7]. Overweight and obese groups had significantly higher out-of-pocket care expenditures (RMB 2613.60/year and RMB 2768.49/year, respectively) compared to the normal-weight group (RMB 1804.45/year) [8]. Overall, it was estimated that 6.13% of out-of-pocket expenditures were attributable to obesity and 5.18% to overweight in the Chinese population aged 45 and older in 2013 [8]. The World Health Organization (WHO) recognizes obesity as a major global health issue and has been trying to solve it in various ways [9].

Obesity is a complex interaction between multiple genetic, socioeconomic, and cultural factors. The food environment has emerged as a key contributor to obesity [5,10]. The broad concept of food environment consists of the collective physical, economic, policy, and sociocultural surroundings; opportunities; and conditions that influence people’s food and beverage choices and nutritional status [11]. The neighborhood or community food environment is commonly characterized as the number, type, location, and accessibility of multiple settings where people eat or procure food, such as supermarkets, convenience stores, fast-food restaurants, and full-service restaurants and the availability, affordability, and acceptability of foods in a community’s food outlets [12,13]. Many methods have been used in the measurement of the neighborhood food environment, such as a questionnaire or phone survey [14,15,16], commercial data list [17,18], and Geographic Information System (GIS) Technology [19,20,21]. The Baidu Map Application Programming Interface (API) (Baidu, Beijing, China) has been widely used in fields such as transportation, surveying, and mapping engineering [22,23], and has been proved effective in studies of obesogenic environments either [24].

Globally, a growing body of evidence has drawn attention to the associations between the neighborhood food environment and BMI [25]. In these studies, associations between density/proximity measures of food outlets and BMI were investigated [25]. However, some results of studies have been inconsistent, with associations for the same type of relationship being positive, negative, or nonexistent [26,27]. Cobb et al. reviewed 71 studies in the USA and Canada and found that the association between food outlet availability and obesity were predominantly null; among non-null associations, there was a trend toward inverse associations between supermarket availability and obesity and direct associations between fast food and obesity in adults [25]. Gamba et al. reviewed 51 studies that analyzed the relationship between the community food environment and obesity among Americans of all ages and found that 80% of studies reported at least one significant result [28]. Holsten reviewed community food environment and obesity in the USA and Australia in both children and adults and found mixed results: two of all seven studies did not find any significant association, whereas the other five reported significant results [29]. In a systematic review of 54 US-based studies on neighborhood food environment, Larson et al. concluded that residents of neighborhoods with better access to supermarkets and limited access to convenience stores and fast-food outlets tend to have healthier diets as well as lower risks of obesity [30]. However, a study conducted in Japan found that better access to supermarkets was related to higher BMI [31], and another research among community-dwelling older adults in New Jersey found that supermarkets were not associated with obesity [32]. This inconsistency indicates that findings from different cities and countries may not be directly comparable because of differences in urban density, legislation, economic or political contexts, food cultures, and traditions [33,34]. So far, most of the studies on the relationship between food environment and obesity have been conducted in western settings and developed countries, such as North America, the United Kingdom, Australia, and Japan [27,31,35]. However, evidence from middle-income countries such as China is limited. The conditions of the neighborhood food environment between developed countries and developing countries are different, and so are people’s dietary habits [36]. Findings from developed countries should not be directly transferable to the developing countries. Therefore, more evidences from middle-income countries need to be provided.

Since the market reforms in 1978, China’s economy has experienced unprecedented rapid development, residents’ income has increased, and a modern food system has been gradually established. In the late 1990s, supermarkets, western fast-food restaurants, and convenience stores have entered some cities in China. Changes in income level and environment have largely affected the dietary behavior and nutritional status of Chinese residents, and Chinese residents are undergoing a process of nutrition transition [37,38]. The proportion of Chinese adult residents with out-of-home eating behavior increased from 14.6% in 2002 to 20.2% in 2010–2012 [39], and the rate increased to 45.1% in 2015 [40]. The energy contribution from out-of-home eating among adults from 1991 to 2011 increased from 8.6% to 16.5% [41]. Although the rate of out-of-home eating among the elderly in China was lower than that of young and middle-aged adults, it was still rising. From 2010 to 2012, the rate of out-of-home eating among the elderly in China was 5.6%, and the rate in big cities was 13.5% [39]. A study in Beijing found that 15.1% of the elderly had the habit of out-of-home eating [42]. In Tianjin, the proportion of elder people who eat out was 10.0% [42]. Among Chinese restaurants, Chinese fast-food restaurants were the most popular choice for the elderly when dining out, and modern restaurants, such as bakeries/cafes and other restaurants (Japanese restaurants, etc.), were less popular [43,44].

Research on the relationship between the food environment and obesity is still in its infancy in China, and the number of studies is relatively small. A review found that 10 out of 11 studies reported a significant association, whereas the remaining study reported a null relationship [36]. However, studies in China mostly use subjective measurement methods rather than objective measurement methods when evaluating the food environment, which easily led to recall bias and social expectation bias [36,45].

The influence of the food environment may be highly specific to the nutritional habits of particular subgroups. As a result of decreased mobility, the dietary habit and weight status of older adults may be more influenced by their neighborhoods compared to school-aged children, who are influenced by both home and school food environments, and the working-age population who are influenced by both home and worksite food environments [46]. Research on the association between the food environment and BMI has so far focused on children and average adults, and research on older adults has been scarce. However, China and many countries in the world are in an aging society. China has been an aging society since 2000. At the end of 2019, the population of those over 60-year-olds in China reached 254 million, accounting for 18.1%, and the population of those over 65 years old reached 176 million, accounting for 12.6% [47]. Therefore, focusing on the health of older adults is especially important. The mixed findings, as well as the lack of Chinese-based evidence and older adults’ evidence, suggest that more studies on the associations between the neighborhood food environment and obesity are needed.

In the present study, we examined the association between neighborhood food environment and BMI among older adults in Beijing, China. The objectives of this study are, first, to clarify the association between different types of food outlets and BMI in the elderly; second, to identify the key types of food outlets that affect the BMI of the older adults; and finally, to make recommendations to improve the neighborhood food environment, thereby improving the BMI and health of the elderly.

## 2. Materials and Methods

### 2.1. Study Design

A cross-sectional study design was used to examine the food environments of urban and suburban elderly adults in Beijing, China.

### 2.2. Participants

Our analyses were based on a cross-sectional survey on the dietary behavior and influencing factors of the elderly conducted in 2019. Participants were recruited from 12 communities of three districts in Beijing, China. In each community, with the help of the community staff, we invited qualified older adults to participate in the survey by phone or door-to-door. A total of 1800 older adults were invited, and 1775 responded and participated in this survey. The response rate was 98.6%. Valid questionnaires (Table S1) were restricted to participants who provided complete information on age, gender, marital status, and whose height and weight were measured correctly. There were a total of 1764 valid questionnaires, and the efficiency of the questionnaire was 99.4%.

The inclusion criteria included: 65–80 years old, retired from work, community-dwelling, living in only one community for more than 2 years; functionally independent.

The exclusion criteria were participants who were unable to eat normally or were cognitively impaired.

### 2.3. Sample

#### 2.3.1. Sampling Method

In this study, a multi-stage stratified random sampling method was used. First, three districts (Haidian District, Shunyi District and Miyun District) were selected as the target districts for this survey, which represented different economic levels and geographic locations of Beijing: Haidian District was the closest to the center of Beijing, with the highest economic level, followed by Shunyi District and finally Miyun District; second, an urban street and a suburban street in each district were selected; third, two communities in each street were selected; lastly, older adults in each community were randomly selected.

#### 2.3.2. Sample Size Calculation

For the calculation of the sample size, the variable used was the obesity rate of older adults in China [1,48]. Using the following formula, the sample size was calculated: N = Z_1-α/2_^2^ *p*(1 − *p*)/e^2^. Among these parameters, α = 0.05, Z_1-α/2_ = 1.96, e = 0.03; *p* = 0.15 indicated the obesity rate of older adults in China. A total of three districts were investigated, and a 10% dropout rate was taken into account to obtain the final sample size. For validity, 1800 older adults were needed.

### 2.4. Ethical Review

The study protocol was reviewed and approved by the Peking University Biomedical Ethics Committee. The ethical approval project identification code is IRB00001052-17112. The study was carried out according to the principles of the Declaration of Helsinki. All participants read the informed consent form, voluntarily agreed to participate in this study, and signed the informed consent form before the study. Written informed consent was obtained from each participant before enrolment in the study and then preserved by researchers.

### 2.5. Participants’ Basic Information

The participants’ basic information was collected through questionnaires, including address, age, gender, marital status, education level, income level, frequency of exercise, and frequency of smoking and drinking. The questionnaire was filled out one by one by the investigators and the participants. Basic information was self-reported by participants. Neighborhood socioeconomic level was determined by the investigator based on documents from the National Bureau of Statistics [49].

### 2.6. Physical Measurement

Participants’ height and weight were measured twice to the nearest 0.1 cm and 0.1 kg by trained investigators following standardized procedures with a height–weight meter (RGZ-160; Suheng, Jiangsu, China).

Height measurement [50]: Participants stood barefoot in an upright posture on the floor of the altimeter. The heel, sacrum, and shoulder blades were in contact with the pedestal of the altimeter. The head was upright, and the eyes were straight ahead. The upper edge of the tragus and the lower edge of the orbit were on the same horizontal line. The record was in centimeters with one decimal place.

Weight measurement [50]: Participants tried to remove their clothes as much as possible and stand naturally at the center of the scale. The data were read after participants stood firm. The record was in kilograms with one decimal place.

### 2.7. Neighborhood Food Environment Measurement

#### 2.7.1. Usage of Baidu Map API

We used Baidu Map API to get information about food outlets around the community, including names and coordinates [51]. The flow process of using the Baidu Map API [24] was as follows: (1) Extract the community coordinates, call the Geocoding API, and enter the community address to obtain the coordinate. (2) Extract POI (point of interest) coordinates within the target radius: call the Place API, use the circular area search method, enter the community coordinates and POI keywords (supermarkets, stores, convivence stores, restaurants, etc.), and obtain the POI names and coordinates within the target radius (1000 m) around the community; (3) Filter the POI information within the distance of the target walking path: call the Direction API, use the walking query retrieval service, enter the coordinates of the community and its surroundings, obtain the walking path distance, and filter the POI information within the target walking path distance (250 m, 500 m, 800 m, 1000 m). These distances were based on the following facts [52,53]: the space within 250 m (about 5 min walking distance) from home is the most frequent place for older adults in daily life; the space within 500 m from home is the main shopping area for older adults, which is about 10 min walking distance; only about 10% of older adults will buy food beyond 1000 m. Afterward, we were able to obtain a list of food outlets.

#### 2.7.2. Classification of Food Outlets

The food outlets were classified into different types. Study investigators used knowledge of the local area food chains coupled with the National Economic Industry Classification (GB T 4754-2017) [54], Tian Yancha [55], and Dianping [56] to refine the list mentioned previously to different types. Tian Yancha is a website for querying corporate information, including industrial and commercial information, and the information is accurate and reliable. Tian Yancha data come from the National Enterprise Credit Information Disclosure System, China Judgment Documents Network, China Enforcement Information Open Network, State Intellectual Property Office, Trademark Office, Copyright Office, and other authoritative websites. Dianping is a website offering local business search, user-generated reviews, detailed business information, and other merchant services. The definition of different types of food establishments was based on National Economic Industry Classification. Then, we divided all food outlets into different types by checking their business scope in Tian Yancha and Dianping. The list mentioned previously was refined to the following categories: (1) supermarket (comprehensive retail activities in supermarkets that sell fresh food, processed food, pre-packaged food, and daily necessities, etc.); (2) convenience store (retail activities in the form of small supermarkets to meet the proportional needs of customers as the main purpose, such as convenience stores and small grocery stores with limited selection); (3) greengrocer (retail activities specializing in fresh vegetables and fruits); (4) sit-down restaurant (catering activities that provide a variety of dishes for lunch and dinner in a certain place, and deliver meals by the waiter, such as Chinese dinners, western dinners, hot pots, etc.); (5) Chinese fast-food restaurant (fast and convenient Chinese catering services in a certain place or through specific equipment, such as Chinese set meals, fried rice, noodles, steamed buns, local cuisines, etc.); (6) western fast-food restaurant (fast and convenient western catering services in a certain place or through specific equipment, such as McDonalds and Kentucky Fried Chicken, pizzerias, limited-service facilities, such as Subway, etc.). Some food outlets present in the neighborhoods were excluded from the study because they did not fit the classifications used, such as bars, coffee shops, dessert shops, etc.

### 2.8. Variables

#### 2.8.1. Body Mass Index

After calculating the average height and weight, BMI (kg/m^2^) was calculated to describe the sample.

#### 2.8.2. Neighborhood Food Environment Variables

The density of the food outlets was measured by the number of the six types of food outlets within each walking distance buffer zone (250 m, 500 m, 800 m, 1000 m), so we had 24 food environment variables. If a store was both a convenience store and a greengrocer, it was categorized as both a convenience store and greengrocer.

Proximity to food outlets can be measured by the nearest distance to the six types of food outlets. If no food outlet was found within the target circular buffer zone (1000 m), a larger circular buffer zone was searched.

#### 2.8.3. Confounders

Confounders in our study were determined through literature review [31] and expert discussions, including individual-level sociodemographic characteristics, such as age, gender, marital status, education level, income level; behavioral factors, such as frequency of exercise, frequency of smoking and drinking, and economic level of the communities.

### 2.9. Statistical Analysis

Statistical analyses were performed using SPSS Statistics 20.0 (IBM Corp., Armonk, NY, USA). Participant and food environment characteristics were examined with descriptive statistics. The characteristics of the participants were expressed in frequency and percentage. Participants’ neighborhood food environment characteristics were expressed by mean and standard deviation. Multiple linear regression analysis was performed on the association between BMI and each food environment variable. In Model 1, only the food environment variables were included. To adjust for potential confounders, demographic characteristics (age, gender, marital status, education level, income level), neighborhood socioeconomic level (urban or suburban), and behavioral factors (drinking, smoking, and frequency of exercise) were included in Model 2. *p* < 0.05 was considered statistically significant.

## 3. Results

### 3.1. Participants Characteristics

Participants (*n* = 1764) had a mean age of 69.7 years (±4.32) and an average BMI of 26.3 kg/m^2^ (±3.50). Over 60% of the elderly were overweight and obese. The sex ratio of men and women was about 1/1.4. The number of elderly from urban and suburban areas was approximately the same. Only 17.0% of the elderly had a habit of smoking, and 75.1% drank less than once a week. More details of individual characteristics are shown in Table 1.

### 3.2. Neighborhood Food Environment Characteristics

**Figure 1:**
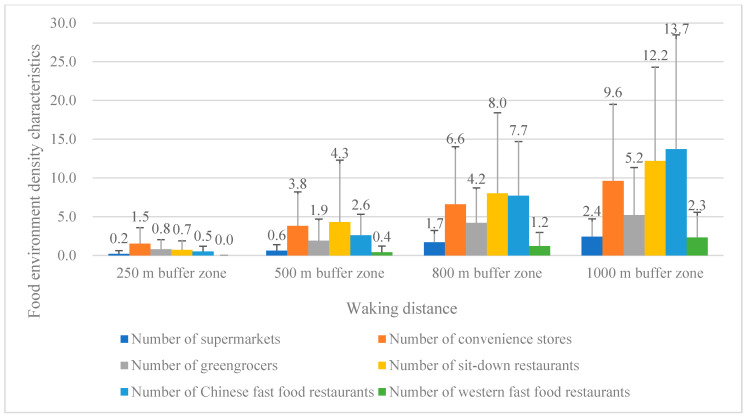
Participants’ neighborhood food environment density characteristics.

**Figure 2:**
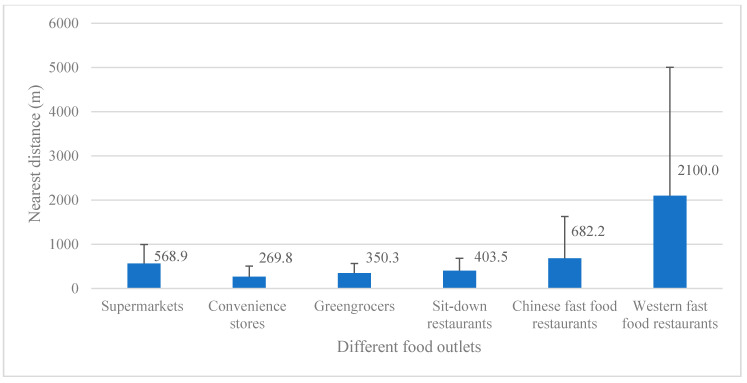
Participants’ neighborhood food environment proximity characteristics.

Among the three types of stores, convenience stores had the best access, with the most number and nearest distance, followed by greengrocers and supermarkets. Among these three types of restaurants, sit-down restaurants had the easiest access in most buffer zones, followed by Chinese fast-food restaurants and western fast-food restaurants. Among all types of food outlets, western fast-food restaurants had the worst access, whose nearest distance of two communities even reached more than 8000 m. Detailed information on participants’ neighborhood food environment density characteristics and proximity characteristics is shown in Table 2/Figure 1 and Table 3/Figure 2.

### 3.3. Association between Neighborhood Food Environment and BMI

Table 4 shows the results of the regression analyses of neighborhood food environment density characteristics and BMI for Model 1 and Model 2. In total, 23 food environment variables (five/six types of stores and four buffer zones) were analyzed for their possible association with BMI. Within the 250 m buffer zone, those who live in areas with more greengrocers and sit-down restaurants tended to have higher BMI. One additional greengrocer in the neighborhood (within 250 m buffer zone) was associated with a 0.281-point increase in BMI (*p* < 0.001), and one additional sit-down restaurant in the neighborhood (within 250 m buffer zone) was associated with a 0.304-point increase in BMI (*p* < 0.001), after controlling for potential confounders. Within the 500 m buffer zone, supermarkets were positively associated with BMI, and one additional supermarket in the neighborhood (within 500 m buffer zone) was associated with a 0.593-point increase in BMI (*p* < 0.001) after controlling for potential confounders. Within the 800 m buffer zone, better access to convenience stores tended to be associated with higher BMI, and one additional convenience store in the neighborhood (within 800 m buffer zone) was associated with a 0.057-point increase in BMI (*p* < 0.001) after controlling for potential confounders. Within the 1000 m buffer zone, living with more Chinese fast-food restaurants tended to have higher BMI, one additional Chinese fast-food restaurant in the neighborhood (within 1000 m buffer zone) was associated with a 0.071-point increase in BMI (*p* = 0.001); in contrast, those living with more western fast-food restaurants tended to have lower BMI, and one additional western fast-food restaurant in the neighborhood (within a 1000 m buffer zone) was associated with a 0.400 point decrease in BMI (*p* < 0.001). No direct associations were found between other food environment variables and BMI after controlling for potential confounders.

Table 5 shows the results of the regression analyses of neighborhood food environment proximity characteristics and BMI for Model 1 and Model 2. In total, six proximity variables were analyzed for their possible association with BMI. After controlling for potential confounders, there was a negative association between nearest distance to greengrocers and BMI (β = −0.004, *p* < 0.001), i.e., better access to greengrocers was associated with higher BMI. No direct associations were found between other food environment proximity variables and BMI after controlling for potential confounders.

## 4. Discussion

With socioeconomic development, nutrition-related chronic diseases, such as obesity, have become one of the main public health problems worldwide [58]. In recent years, to control the increasing obesity rate and comprehensively clarify the relationship between obesity and dietary factors, more and more scholars have shifted their research focus from individual-level dietary behavior factors to community-level food environmental factors [59,60]. Our findings indicate that the numbers of supermarkets, convenience stores, greengrocers, sit-down restaurants, and fast-food restaurants (both Chinese and western fast-food restaurants), as well as the nearest distance to greengrocers, were all associated with BMI among older adults in Beijing, China.

Our results showed that the number of greengrocers was positively associated with BMI among older adults in Beijing, China (in 250 m buffer zone). At the same time, we found that there was a negative association between the nearest distance to greengrocers and BMI. In other words, better access to greengrocers was associated with higher BMI. This is contrary to the commonly accepted hypothesis: better access to fresh vegetables and fruits is associated with better dietary quality and lower BMI. This result may be due to Chinese eating habits. Vegetables are an important part of the Chinese diet. The overall vegetables consumption rate among elderly adults in China was 99.5%, and the P50 vegetable intake was 242.3 g/d in 2015 [61]. The elderly have a high frequency of shopping trips, and the proportion of shopping trips 6–7 days per week was 48.7% [62]. Therefore, better access to greengrocers may decrease the walkability of a neighborhood, which has been associated with obesity [63]. For the elderly, the best distance to greengrocers was most likely to be within the range of 600–900 m, and the one-way walking time is about 10–15 min [64]. Physical activity of this intensity had a positive effect on the physical and mental health of the elderly [64].

The greater the number of sit-down restaurants within a 250 m buffer zone, the higher the BMI of the elderly. According to our questionnaire about the eating behavior of the elderly, they usually choose sit-down restaurants closer to home when they eat out. There may be a positive association between out-of-home eating and body weight [65]. Therefore, when the number of sit-down restaurants within a 250 m buffer zone increased, they may eat out more frequently, thereby increasing the risk of being overweight and obese.

We found that participants living in areas with more supermarkets tended to have higher BMI (in 500 m buffer zone). In previous research, associations between supermarkets and BMI were inconsistent. In general, studies in the United States (US) have suggested that better access to supermarkets is related to healthier food intake and lower levels of obesity since supermarkets tend to offer a variety of high-quality products at lower cost [30]. However, a study in Japan found the opposite result, a positive association between supermarket accessibility and BMI [31]. This inconsistency may be caused by different food systems, policies, socioeconomic, and cultures between eastern and western countries. In addition to fresh fruits and vegetables, supermarkets tend to provide more processed food or junk food. Therefore, if people shop in supermarkets, they may consume many processed foods or junk foods, which might cause a gain in body weight. The space within 500 m from home is the main shopping area for older adults [52,53], so when there are more supermarkets within the 500 m buffer zone, the elderly are exposed to more unhealthy foods. This may cause a higher BMI. Our result was opposite to the US and consistent with Japan, suggesting the possible different roles of supermarkets in eastern and western cultures.

Similar to studies in the US, we also found a positive association between convenience stores accessibility and BMI (only in the 800 m buffer zone). The reason is that convenience stores generally offer various types of pre-packaged food, junk food, sugar-sweetened beverages, and so on.

As we predicted, the more Chinese fast-food restaurants, the higher the BMI (only within the 1000 m buffer zone). This is because when people have better access to Chinese fast-food restaurants, they are more likely to eat out. It was somewhat unexpected that better access to western fast-food restaurants was associated with lower BMI (only in 1000 m buffer zone). Interestingly, two previous studies among African–American adults found that greater availability of fast food, pizzerias, limited-service facilities, and bakeries was associated with more desirable dietary characteristics, such as greater fruit, vegetable, and fiber consumption [66,67], which were expected to be healthy eating indicators. In our study, this result may be because Chinese older adults rarely consume western fast food [43,44]. When there are more western fast-food restaurants in the neighborhood, this may reduce the possibility of eating out.

Our study adds to the evidence base for the potential influence of the food environment in China. Although we have obtained some statistically significant results, we cannot conclude whether a certain type of food outlet is healthy or unhealthy. In this study, we only considered the accessibility and availability of food outlets and did not consider other dimensions of the neighborhood food environment, such as affordability, acceptability, and accommodation, nor the consumption environment of food outlets [68]. Furthermore, there is inter-individual variability in how people interact with the food environment [18]. Unmeasured individual, peer, family, and community-level factors, such as personal taste, preferences, family food rules, and consumption environments in food establishments, could also mediate or moderate the relationship between the food environment and BMI. For policymakers, the existing evidence in China is not clear or sufficient and cannot support making decisions on whether to promote or discourage a particular type of food outlet. Therefore, it is necessary to carry out relevant research on different dimensions of the food environment, especially experimental research, to draw causal conclusions. At the same time, strategies that promote availability, accessibility, and affordability of healthy foods must be implemented across the food system [69]. It is worth noting that interventions that target food environments and food systems are frequently and systematically undermined by the coordinated efforts of powerful food and beverage industry groups [69]. This will be a key issue for policymakers to consider in the future.

Our study has a number of notable strengths related to innovation, exposure and outcome measurements, study design, and statistical analysis [25]. Innovation concerns are as follows. (1) To our knowledge, this was the first study to investigate such associations among Chinese older adults. Our research provides evidence of the association between the food environment and BMI among older adults in China, and it can also provide a scientific basis for Chinese policymakers. (2) We used the new method of Baidu Map API to study the neighborhood food environment, and this may provide new ideas for the methodology of related research in the future. Previous methods of measuring the food environment have certain disadvantages. For example, the accuracy of data from commercial lists or provided by government agencies has been questioned [70,71]. As for geolocation, Geographic Information System (GIS) is widely used at present [68]. However, research based on the GIS method faces challenges about data quality and geolocation accuracy [72]. The traditional GIS platform also has the shortcoming of being a complex process and high-cost. Compared with the traditional GIS platform, Baidu Map API has the advantages of convenient development, low investment cost, and good performance [24]. Baidu Map API has been widely used in transportation and surveying engineering fields and has been proved effective in the study of food environment: the positive predictive values for fast-food restaurants and convenience stores were 0.84 and 0.86, respectively [24]. Exposure and outcome concerns included the following. (3) In our study, participants’ height and weight were measured by researchers at the survey site; therefore, BMI was more accurate than self-reported. (4) Our characterization of the neighborhood environment was based on walking distances (as opposed to circular buffers), which showed the actual distances that people must travel to access food, producing a more accurate representation of accessibility to food outlets because even a small circular buffer (0.25 miles or 400 m) may include outlets that residents consider to be outside walking distance. Despite the widespread use of the term “neighborhood food environment”, there has been no consensus on what this term means in relation to a geographic area [73]. The neighborhood food environment could be measured based on circular buffers or street networks, but if there is no information on the actual locations of respondents, respondents could only be grouped into larger geographic areas. Design concerns included the following. (5) There were no restrictions on the diseased/obese participants, preventing selection bias, and (6) a multi-stage stratified random sampling method was used in this study to solve neighborhood self-selection bias. Analysis concerns included the following. (7) Many confounding factors were included in our model—not only demographic characteristics, such as age, gender, marital status, education level, and income level, but also neighborhood socioeconomic level and behavior characteristics, such as drinking, smoking, and frequency of exercise.

Our study is subject to a few limitations. First, our study area was limited to Beijing, which may not be representative of China. Although we adopted a multi-stage stratified random sampling method, sampling from different economic levels and geographic locations in Beijing, it can basically represent the situation in Beijing. However, since Beijing is a city with a high economic level in China, it does not represent the overall situation of China, so our findings need to be replicated in other areas, particularly in cities of different economic levels. Second, we did not control for variables on the causal pathway. Incorporating variables that are more directly related to BMI (e.g., dietary intake) should be considered in future studies. Third, food outlet data were not validated in person. Finally, like many studies in the area, the current work used a cross-sectional design, and causal inferences should not be made.

## 5. Conclusions

Our study finds some significant associations between neighborhood food environment and obesity, which adds to the evidence base for such relationships in China. However, the current results are not strong enough to conclude whether a certain type of outlet is healthy or unhealthy. Policymakers will need to rely on more evidence to derive concrete policy recommendations. In future studies, a bigger picture of food environments should be taken into account. It is necessary to carry out relevant research on different dimensions of the food environment, especially experimental research, to draw causal conclusions.

## Figures and Tables

**Table 1:** Characteristics of the participants (*n*, %).

Items	*n*	%
BMI (kg/m^2^) ^1^		
Underweight: <18.5	7	0.4
Normal: 18.5–24.9	638	36.2
Overweight: 25–29.9	620	35.1
Obese: ≥30	499	28.3
Gender		
Male	730	41.4
Female	1034	58.6
Age		
65–69	983	55.7
70–74	483	27.4
75–79	298	16.9
Marital status		
Unmarried	3	0.2
Married	1482	84.0
Widowed	260	14.7
Divorced or separated	19	1.1
Education level		
≥Bachelor’s degree	190	10.8
Middle school ^2^	923	52.3
≤Primary school	651	36.9
Income level (RMB) ^3^		
≤2000	409	23.2
2000–3500	652	37.0
3500–5000	376	21.3
5000–10,000	223	12.6
≥10,000	24	1.4
Missing	80	4.5
Neighborhood socioeconomic level		
Urban	899	51.0
Suburban	865	49.0
Frequency of exercise		
Never	198	11.2
1–2/week	55	3.1
3–4/week	83	4.7
5–6/week	35	2.0
Everyday	1387	78.6
Missing	6	0.3
Smoking ^4^		
No	1464	83.0
Yes	300	17.0
Drinking		
≥Once a week	436	24.7
<Once a week	1325	75.1
Missing	3	0.2

Note. ^1^ Body mass index: a measurement of someone’s weight in relation to their height. ^2^ Including high school, junior high school, technical secondary school, professional technical school, etc. ^3^ Monthly household per capita income in renminbi (RMB): ≤2000 (low level), 2000–3500 (low to medium level), 3500–5000 (medium level), 5000–10,000 (medium to high level), ≥10,000 (high level) [57] ^4^ No: never smoked or has quit smoking; Yes: still smoking or has failed to quit smoking.

**Table 2:** Participants’ neighborhood food environment density characteristics.

Numbers of Food Outlets	250 m Buffer Zone		500 m Buffer Zone		800 m Buffer Zone		1000 m Buffer Zone	
	Mean	SD	Mean	SD	Mean	SD	Mean	SD
Number of supermarkets	0.2	0.4	0.6	0.8	1.7	1.5	2.4	2.3
Number of convenience stores	1.5	2.1	3.8	4.4	6.6	7.4	9.6	9.9
Number of greengrocers	0.8	1.2	1.9	2.8	4.2	4.5	5.2	6.1
Number of sit-down restaurants	0.7	1.2	4.3	8.0	8.0	10.4	12.2	12.1
Number of Chinese fast-food restaurants	0.5	0.7	2.6	2.7	7.7	7.0	13.7	14.8
Number of western fast-food restaurants	0.0	0.0	0.4	0.8	1.2	1.8	2.3	3.3

Note. SD: standard deviation.

**Table 3:** Participants’ neighborhood food environment proximity characteristics.

Nearest Distance to Food Outlets (m)	Mean	SD	Minimum	Maximum
Nearest distance to supermarkets (m)	568.9	428.2	91.0	1676.0
Nearest distance to convenience stores (m)	269.8	240.1	0.1	825.0
Nearest distance to greengrocers (m)	350.3	218.0	77.0	705.0
Nearest distance to sit-down restaurants (m)	403.5	279.7	52.0	992.0
Nearest distance to Chinese fast-food restaurants (m)	682.2	947.0	169.0	3563.0
Nearest distance to western fast-food restaurants (m)	2100.0	2901.4	339.0	8332.0

Note. The distance is from the food outlet to the neighborhood. SD: standard deviation.

**Table 4:** Associations between the density of food outlets and body mass index.

Number of Food Outlets within Different Buffer Zones	Model 1 ^1^			Model 2 ^2^		
	β	SE	*p*	β	SE	*p*
**Within 250 m buffer** **zone**						
Number of supermarkets	−0.001	0.030	0.973	0.011	0.036	0.763
Number of convenience stores	−0.060	0.036	0.096	−0.044	0.037	0.230
Number of greengrocers	0.326	0.072	<0.001	0.281	0.072	<0.001
Number of sit-down restaurants	0.298	0.073	<0.001	0.304	0.072	<0.001
Number of Chinese fast-food restaurants	−0.029	0.031	0.355	−0.026	0.033	0.427
**Within 500 m buffer zone**						
Number of supermarkets	0.603	0.108	<0.001	0.593	0.108	<0.001
Number of convenience stores	−0.018	0.056	0.747	−0.008	0.055	0.884
Number of greengrocers	0.063	0.045	0.166	0.046	0.046	0.313
Number of sit-down restaurants	0.010	0.025	0.686	0.017	0.026	0.515
Number of Chinese fast-food restaurants	−0.024	0.027	0.377	−0.010	0.028	0.723
Number of western fast-food restaurants	0.004	0.022	0.856	0.015	0.025	0.551
**Within 800 m buffer zone**						
Number of supermarkets	0.049	0.054	0.367	0.073	0.054	0.179
Number of convenience stores	0.057	0.012	<0.001	0.057	0.012	<0.001
Number of greengrocers	0.048	0.067	0.473	0.033	0.067	0.622
Number of sit-down restaurants	0.026	0.025	0.299	0.033	0.026	0.203
Number of Chinese fast-food restaurants	−0.003	0.025	0.904	0.008	0.026	0.756
Number of western fast-food restaurants	−0.038	0.023	0.105	−0.028	0.025	0.262
**Within 1000 m buffer zone**						
Number of supermarkets	0.003	0.038	0.938	−0.023	0.042	0.582
Number of convenience stores	0.036	0.034	0.293	0.022	0.037	0.549
Number of greengrocers	0.036	0.034	0.284	0.008	0.037	0.829
Number of sit-down restaurants	−0.028	0.041	0.500	−0.021	0.040	0.602
Number of Chinese fast-food restaurants	0.102	0.017	<0.001	0.071	0.021	0.001
Number of western fast-food restaurants	−0.497	0.079	<0.001	−0.400	0.084	<0.001

Note. SE: standard error. ^1^ Non-adjusted model. ^2^ Adjusted for age, gender, marital status, educational attainment, income level, neighborhood socioeconomic level, drinking, smoking, and frequency of exercise. Results are presented without associated covariate information for conciseness.

**Table 5:** Associations between proximity to food outlets and body mass index.

Nearest Distance to Food Outlets	Model 1 ^1^			Model 2 ^2^		
	β	SE	*p*	β	SE	*p*
Nearest distance to supermarkets	0.119	0.079	0.132	0.099	0.083	0.233
Nearest distance to convenience stores	0.006	0.028	0.830	−0.033	0.031	0.287
Nearest distance to greengrocers	−0.004	0.001	<0.001	−0.004	0.001	<0.001
Nearest distance to sit-down restaurants	−0.004	0.028	0.884	−0.028	0.030	0.356
Nearest distance to Chinese fast-food restaurants	−0.007	0.030	0.813	0.046	0.034	0.175
Nearest distance to western fast-food restaurants	−0.048	0.029	0.103	−0.014	0.033	0.670

Note. SE: standard error. ^1^ Non-adjusted model. ^2^ Adjusted for age, gender, marital status, education level, income level, neighborhood socioeconomic level, drinking, drinking, smoking, and frequency of exercise. Results are presented without associated covariate information for conciseness.

## Supplementary Materials

The following are available online at https://www.mdpi.com/1660-4601/17/20/7658/s1, Table S1: Basic Information Questionnaire.
