# Supplementary material for: Association between Neighborhood Food Environment and Body Mass Index among Older Adults in Beijing, China: A Cross-Sectional Study
Source: Int J Environ Res Public Health. 2020 Oct 20;17(20):7658. doi: 10.3390/ijerph17207658 (PMC7589694; doi:10.3390/ijerph17207658)
Supplement: Supplementary file 1 [file ijerph-17-07658-s001.pdf]

Table S1. Basic Information Questionnaire.

Hello, dear residents!
Please answer the following questions independently and truthfully. There is no right or wrong answer, each of your answers is very important to us. At the same time, we will keep you confidential, please fill in truthfully according to your own real thoughts. Thank you for your cooperation!

1. Your address: _____District _____Street _____Community
2. Your gender: ①Male ②Female
3. Your age: _____ years old
4. Height: _____ meters
5. Weight: _____ kg
6. Your highest education level:
   ① Master's degree and above ② University or college
   ③ Secondary technical school, vocational school ④ High school ⑤ Junior high school
   ⑥ Elementary school ⑦ Did not graduate from elementary school ⑧ Did not go to school
7. Your marital status:
   ① unmarried ② married ③ widowed ④ divorced or separated
8. What is the monthly income per capita in RMB (after tax) of your family?
   ① Below 2000 ② 2000–3500 ③ 3500–5000
   ④ 5000–6500 ⑤ 6500–10,000 ⑥ 10,000 and above
9. In the past month, how often did you exercise? (Walking, running, dancing, ball games, etc.)
   ① Never ② 1–2 times a week ③ 3–4 times a week ④ 5–6 times a week ⑤ every day
10. Do you smoke?
    ① Never smoked ② keep smoking, never tried to quit smoking
    ③ have failed to quit smoking ④ have quit smoking
11. How often did you drink alcohol in the past year?
    ① Drink almost every day ② 3–4 times a week ③ 1–2 times a week
    ④ 1–3 times a month ⑤ Less than once a month ⑥ Never
